# Supplementary material for: A histological analysis of coloration in the Peruvian mimic poison frog (Ranitomeya imitator)
Source: PeerJ. 2023 Jun 30;11:e15533. doi: 10.7717/peerj.15533 (PMC10317021; doi:10.7717/peerj.15533)
Supplement: Supplemental Information 1 — Supplemental Table 1. Reagents used in histological processing and staining. Tissue processing reagents were specified by Amato et al. (2018). Staining reagents were specified by Newcomer’s Schmorl Melanin Staining Protocol and exposure times were modified for optimal staining of R. imitator tissue. [file peerj-11-15533-s001.docx]

Supplemental Table 1. Reagents used in histological processing and staining. Tissue processing reagents were specified by Amato et al. 2018. Staining reagents were specified by Newcomer’s Schmorl Melanin Staining Protocol and exposure times were modified for optimal staining of *R. imitator tissue.*

| Tissue Processing | | |
| --- | --- | --- |
| Reagent | **Time in Reagent** | **Purpose of Reagent** |
| 70% ethanol | 2 hrs, 24ºC | dehydrate tissue |
| 80% ethanol | 1 hr, 24ºC |  |
| 95% ethanol | 1 hr, 24ºC |  |
| 95% ethanol | 1 hr, 24ºC |  |
| 100% ethanol | 1 hr, 24ºC |  |
| 100% ethanol | 1 hr, 24ºC |  |
| 100% ethanol | 1 hr, 24ºC |  |
| Slidebrite | 1 hr, 24ºC | clear tissue |
| Slidebrite | 1 hr, 24ºC |  |
| Slidebrite | 1 hr, 24ºC |  |
| Ultraffin X | 1 hr, 55ºC | embed tissue in wax for sectioning |
| Ultraffin X | 1 hr, 55ºC |  |
| Ultraffin X | 2 hr, 55ºC (under vacuum) |  |
| Staining | | |
| Reagent | **Time in Reagent** | **Purpose of Reagent** |
| Slidebrite | 3 minutes, 3 changes | deparaffinize tissue sections |
| 100% ethanol | 10 dips (~1 min), 2 changes | re-hydrate tissue |
| 95% ethanol | 10 dips (~1 min), 2 changes | re-hydrate tissue |
| Distilled water | 15 seconds | rinse away any debris or residue |
| Ferricyanide solution  (10 mL 1% potassium ferricyanide: 30 mL 1% ferric chloride) | 1.5 minutes | stain melanin deposits black |
| Distilled water | 15 seconds | rinse away excess ferricyanide solution |
| Nuclear fast red solution | 5 minutes | stain cell nuclei red and surrounding tissue pink |
| Distilled water | 15 seconds | rinse away excess nuclear fast red |
| 95% ethanol | 2 dips | dehydrate tissue |
| 100% ethanol | 2 dips | dehydrate tissue |
| Slidebrite | 10 dips (~1 min), 3 changes | clear any non-specific stains |
| Permount® mounting medium | indefinite | fix stains in tissue and adhere cover slip to slide |
