# Supplementary material for: A histological analysis of coloration in the Peruvian mimic poison frog (Ranitomeya imitator)
Source: PeerJ. 2023 Jun 30;11:e15533. doi: 10.7717/peerj.15533 (PMC10317021; doi:10.7717/peerj.15533)
Supplement: Supplemental Information 2 — One-way ANOVA and Tukey’s Studentized Range (HSD) test for the abundance of xanthophores (calculated as total area of xanthophores divided by total area of skin section) found in yellow (striped) and orange (banded and varadero) skin tissue. [file peerj-11-15533-s002.docx]

Supplemental Table 2.  Summary of the distribution of skin sections imaged and analyzed across mimetic morphs, individual frogs, and skin colors.

| **S2. Summary of Histological Sample Distribution** | | | |
| --- | --- | --- | --- |
| **Morph** | **Frog ID** | **Skin Color** | **Skin Sections Analyzed** |
| striped | PO16 | black | 41 |
|  |  | yellow | 0 |
|  |  | green | 11 |
|  | PC22 | black | 67 |
|  |  | yellow | 30 |
|  |  | green | 0 |
|  | PC23 | black | 50 |
|  |  | yellow | 73 |
|  |  | green | 35 |
|  | PC24 | black | 71 |
|  |  | yellow | 42 |
|  |  | green | 10 |
|  | PC25 | black | 62 |
|  |  | yellow | 44 |
|  |  | green | 55 |
|  | PC26 | black | 33 |
|  |  | yellow | 6 |
|  |  | green | 51 |
|  | **TOTAL** black skin sections analyzed = 324 | | |
|  | **TOTAL** yellow skin sections analyzed = 195 | | |
|  | **TOTAL** green skin sections analyzed = 162 | | |
| spotted | TU04 | black | 68 |
|  |  | green | 42 |
|  | TU05 | black | 73 |
|  |  | green | 30 |
|  | TU06 | black | 22 |
|  |  | green | 14 |
|  | 3701 | black | 76 |
|  |  | green | 39 |
|  | 3703 | black | 58 |
|  |  | green | 12 |
|  | 3704 | black | 91 |
|  |  | green | 21 |
|  | **TOTAL** black skin sections analyzed = 388 | | |
|  | **TOTAL** green skin sections analyzed = 158 | | |
| banded | SA01 | black | 63 |
|  |  | orange | 10 |
|  | SA02 | black | 55 |
|  |  | orange | 0 |
|  | SA03 | black | 42 |
|  |  | orange | 74 |
|  | SA04 | black | 52 |
|  |  | orange | 87 |
|  | SA05 | black | 33 |
|  |  | orange | 14 |
|  | SA06 | black | 47 |
|  |  | orange | 23 |
|  | **TOTAL** black skin sections analyzed = 292 | | |
|  | **TOTAL** orange skin sections analyzed = 208 | | |
| varadero | VA01 | black | 54 |
|  |  | orange | 36 |
|  | VA02 | black | 69 |
|  |  | orange | 66 |
|  | VA04 | black | 12 |
|  |  | orange | 55 |
|  | VA05 | black | 77 |
|  |  | orange | 33 |
|  | VA06 | black | 68 |
|  |  | orange | 52 |
|  | VA07 | black | 64 |
|  |  | orange | 0 |
|  | **TOTAL** black skin sections analyzed = 344 | | |
|  | **TOTAL** orange skin sections analyzed = 242 | | |
