# Supplementary material for: A histological analysis of coloration in the Peruvian mimic poison frog (Ranitomeya imitator)
Source: PeerJ. 2023 Jun 30;11:e15533. doi: 10.7717/peerj.15533 (PMC10317021; doi:10.7717/peerj.15533)
Supplement: Supplemental Information 3 — One-way ANOVA test for the coverage of melanophores (calculated as total area of melanophores divided by total area of skin section) found in black skin tissue. [file peerj-11-15533-s003.docx]

Supplemental Table 3. One-way ANOVA test for the coverage of melanophores (calculated as total area of melanophores divided by total area of skin section) found in black skin tissue.

| S3. Melanophore Coverage in Black Skin Tissue | | | | | |
| --- | --- | --- | --- | --- | --- |
| Morph | | **% Melanophores** | **Variance** | **Sample size** | |
| striped | | 17.1500 | 3.861 | 6 frogs, 324 images | |
| spotted | | 16.9333 | 1.510 | 6 frogs, 388 images | |
| banded | | 16.6417 | 3.272 | 6 frogs, 292 images | |
| varadero | | 15.1317 | 0.477 | 6 frogs, 344 images | |
|  | | | | | |
| A one-way ANOVA test with 3 degrees of freedom produced an F-value of 2.190 and a P_r_ > F of 0.1209. | | | | | |
