# Supplementary material for: A histological analysis of coloration in the Peruvian mimic poison frog (Ranitomeya imitator)
Source: PeerJ. 2023 Jun 30;11:e15533. doi: 10.7717/peerj.15533 (PMC10317021; doi:10.7717/peerj.15533)
Supplement: Supplemental Information 4 — One-way ANOVA and Tukey’s Studentized Range (HSD) test for the abundance of xanthophores (calculated as total area of xanthophores divided by total area of skin section) found in green (striped and spotted) and orange (banded and varadero) skin tissue. [file peerj-11-15533-s004.docx]

Supplemental Table 4. One-way ANOVA and Tukey’s Studentized Range (HSD) test for the coverage of xanthophores (calculated as total area of xanthophores divided by total area of skin section) found in yellow (striped) and orange (banded and varadero) skin tissue.

| S4. Xanthophore Coverage in Yellow/Orange Skin Tissue | | | |
| --- | --- | --- | --- |
| Morph | **% Xanthophores** | **Variance** | **Sample Size** |
| banded | 11.385 | 8.898 | 6 frogs, 208 images |
| varadero | 6.445 | 1.946 | 6 frogs, 242 images |
| striped | 6.270 | 2.132 | 6 frogs, 195 images |
|  | | | |
| A one-way ANOVA test with 2 degrees of freedom produced an F-value of 11.7 and a P_r_ > F of 0.0009.  Tukey’s HSD test with an alpha of 0.05 produced a minimum significant difference in mean of 3.1192 and the following results… | | | |
| Morph Comparison | **Difference Between Means** | | **Significance** |
| banded - varadero | 4.940 | | significant |
| banded - striped | 5.115 | | significant |
| varadero - banded | 4.940 | | significant |
| varadero - striped | 0.175 | |  |
| striped - banded | -5.115 | | significant |
| striped - varadero | 0.175 | |  |
