# Supplementary material for: A histological analysis of coloration in the Peruvian mimic poison frog (Ranitomeya imitator)
Source: PeerJ. 2023 Jun 30;11:e15533. doi: 10.7717/peerj.15533 (PMC10317021; doi:10.7717/peerj.15533)
Supplement: Supplemental Information 6 — One-way ANOVA and Tukey’s Studentized Range (HSD) test for the abundance of poison glands (calculated as total area of poison glands divided by total area of skin section) found in the four color morphs of R. imitator. [file peerj-11-15533-s006.docx]

Supplemental Table 6. One-way ANOVA and Tukey’s Studentized Range (HSD) test for the coverage of xanthophores (calculated as total area of xanthophores divided by total area of skin section) found in green (striped and spotted) and orange (banded and varadero) skin tissue.

| S6. Xanthophore Coverage in Green/Orange Skin Tissue | | | |
| --- | --- | --- | --- |
| Morph | **% Xanthophores** | **Variance** | **Sample Size** |
| banded | 11.385 | 8.898 | 6 frogs, 208 images |
| varadero | 6.445 | 1.946 | 6 frogs, 242 images |
| striped | 6.270 | 2.132 | 6 frogs, 162 images |
| spotted | 2.262 | 2.849 | 6 frogs, 158 images |
|  | | | |
| A one-way ANOVA test with 3 degrees of freedom produced an F-value of 21.15 and a P_r_ > F of > 0.0001.  Tukey’s HSD test with an alpha of 0.05 produced a minimum significant difference in mean of 3.2145 and the following results… | | | |
| Morph Comparison | **Difference Between Means** | | **Significance** |
| banded - striped | 5.115 | | significant |
| banded - spotted | 9.123 | | significant |
| banded - varadero | 4.940 | | significant |
| striped - banded | -5.115 | | significant |
| striped - spotted | 4.008 | | significant |
| striped - varadero | -0.175 | |  |
| spotted - banded | -9.123 | | significant |
| spotted - striped | -4.008 | | significant |
| spotted - varadero | -4.138 | | significant |
| varadero - banded | 5.115 | | significant |
| varadero - striped | 0.175 | |  |
| varadero - spotted | 4.138 | | significant |
