# Supplementary material for: A histological analysis of coloration in the Peruvian mimic poison frog (Ranitomeya imitator)
Source: PeerJ. 2023 Jun 30;11:e15533. doi: 10.7717/peerj.15533 (PMC10317021; doi:10.7717/peerj.15533)
Supplement: Supplemental Information 7 — Reagents used in histological staining. Reagents were specified by Newcomer’s Schmorl Melanin Staining Protocol and exposure times were modified for optimal staining of R. imitator tissue. [file peerj-11-15533-s007.docx]

Supplemental Table 7. One-way ANOVA and Tukey’s Studentized Range (HSD) test for the coverage of melanophores (calculated as total area of melanophores divided by total area of skin section) found in green (striped and spotted) and orange (banded and varadero) skin tissue.

| S7. Melanophore Coverage in Green/Orange Skin Tissue | | | | | |
| --- | --- | --- | --- | --- | --- |
| Morph | | **% Melanophores** | **Variance** | **Sample Size** | |
| spotted | | 17.537 | 9.120 | 6 frogs, 158 images | |
| striped | | 12.705 | 5.246 | 6 frogs, 162 images | |
| varadero | | 8.900 | 4.683 | 6 frogs, 242 images | |
| banded | | 6.613 | 6.922 | 6 frogs, 208 images | |
| A one-way ANOVA test with 3 degrees of freedom produced an F-value of 21.11 and a P_r_ > F of > 0.0001.  Tukey’s HSD test with an alpha of 0.05 produced a minimum significant difference in mean of 3.1175 and the following results… | | | | | |
| Morph Comparison | **Difference Between Means** | | | | **Significance** |
| banded - striped | -6.092 | | | | significant |
| banded - spotted | -10.924 | | | | significant |
| banded - varadero | -2.287 | | | |  |
| striped - banded | 6.092 | | | | significant |
| striped - spotted | -4.832 | | | | significant |
| striped - varadero | 3.805 | | | | significant |
| spotted - banded | 10.924 | | | | significant |
| spotted - striped | 4.832 | | | | significant |
| spotted - varadero | 8.637 | | | | significant |
| varadero - banded | 2.287 | | | |  |
| varadero - striped | -3.805 | | | | significant |
| varadero - spotted | -8.637 | | | | significant |
