# Supplementary figures and images for: A histological analysis of coloration in the Peruvian mimic poison frog (Ranitomeya imitator)
Source: PeerJ. 2023 Jun 30;11:e15533. doi: 10.7717/peerj.15533 (PMC10317021; doi:10.7717/peerj.15533)

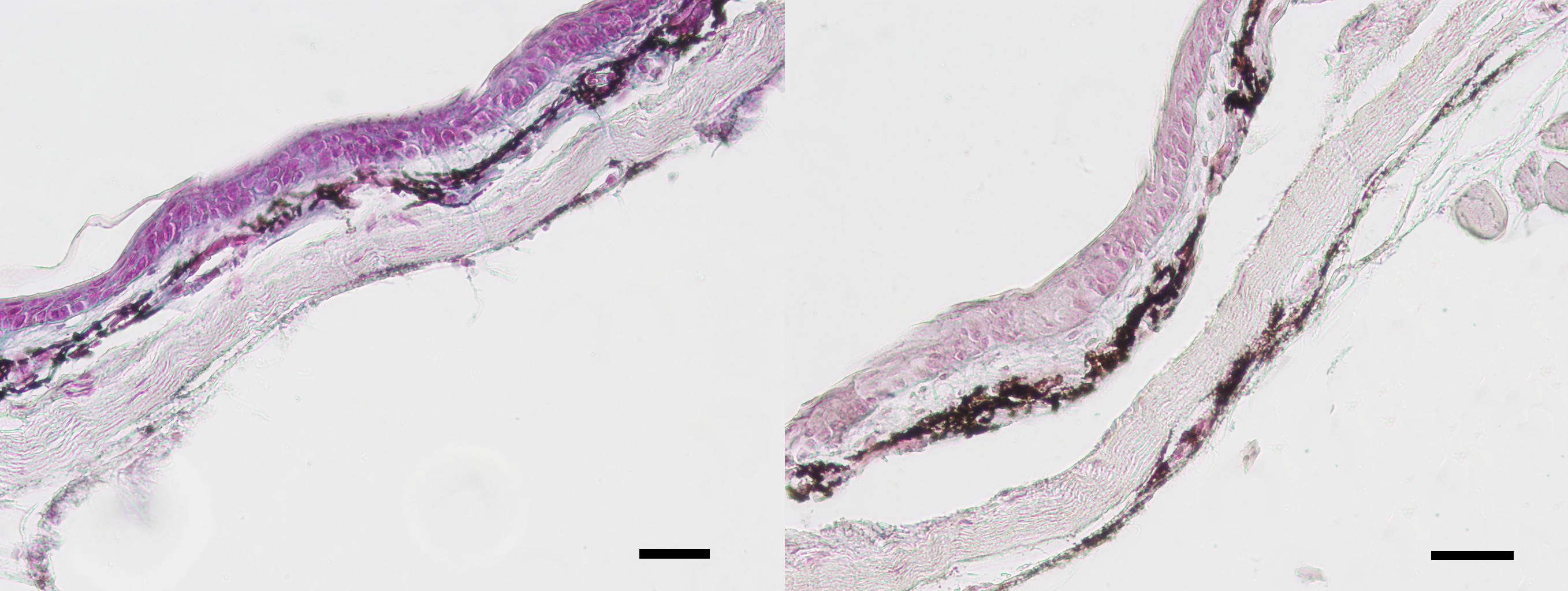

Supplement: Supplemental Information 8 — Magnification level 40×. Scale bar is 100 ųm. [file peerj-11-15533-s008.jpg]
